# Metabotropic glutamate receptor: A new possible therapeutic target for cochlear synaptopathy 

**DOI:** 10.22038/IJBMS.2021.59970.13296

**Published:** 2022-01

**Authors:** Parvane Mahdi, Akram Pourbakht, Alireza Karimi Yazdi, Mahtab Rabbani Anari, Vahid Pirhajati Mahabadi, Mohammad Kamali

**Affiliations:** 1 Department of Audiology, School of Rehabilitation Sciences, Iran University of Medical Sciences. Tehran, Iran; 2 Rehabilitation Research Center, Department of Audiology, School of Rehabilitation Sciences, Iran University of Medical Sciences. Tehran, Iran; 3 Department of Otorhinolaryngology-Head and Neck Surgery, Imam Khomeini Educational Hospital Complex, Tehran University of Medical Sciences. Tehran, Iran; 4 Otorhinolaryngology Research Center, Amir-Alam Educational Complex, Tehran University of Medical Sciences. Tehran, Iran; 5 Department of Neurosciences, School of Medicine. Iran University of Medical Sciences. Tehran, Iran; 6 Department of Rehabilitation Management, School of Rehabilitation Sciences, Iran University of Medical Sciences. Tehran, Iran

**Keywords:** Cochlear synaptopathy, Excitotoxicity, Glutamate, Hidden hearing loss, Noise-induced hearing loss

## Abstract

**Objective(s):**

Cochlear synaptopathy is a common cause of auditory disorders in which glutamate over-activation occurs. Modulating glutamatergic pathways has been proposed to down-regulate post-synaptic excitation.

**Materials and Methods:**

12-guinea pigs as sham and test groups were exposed to a 4-kHz noise at 104 dB SPL, for 2 hr. Pre-exposure intra-tympanic injection with LY354740 and normal saline 9% was applied in the test and sham groups. The amplitude growth of ABR-wave-I and wave-III latency shift with noise were considered in pre- and post-exposure times. The synapses were observed by transmission electron-microscopy.

**Results:**

ABR thresholds recovered 1-week post-exposure in both groups. The reduction of wave-I amplitude at 4, 6, and 8 kHz were statistically different between pre- and 1- day post-exposure and recovered mostly in the sham group. The amount of latency shift in masked ABR was different between pre- and all post-exposure, and the response could not be detected at higher than 50 dB SL noise. However, the response detectability increased to 60 dB SL noise, and the significance of differences between pre- and post-exposure persisted only at the high level of noise in the test group. In electron-microscopy of sham samples, the size of the ribbon was larger, spherical with an irregularity, and hollow. The post-synaptic density was thicker and missed its flat orientation.

**Conclusion:**

The higher slope of the ABR-wave I amplitude, the more tolerance of noise in masked ABR, concomitant with the histological finding that revealed less synaptic damage, confirmed the therapeutic effect of LY354740 in cochlear synaptopathy.

## Introduction

Considering excitatory synapses along with the auditory afferent system, glutamate is recognized as the main neurotransmitter available (1-3). Following the release of glutamate, it exerts the relevant effects through some defined ionotropic (iGlu) and metabotropic glutamate receptors (mGlu) (4, 5). Opposed to iGlu receptors, which relay fast synaptic communication, the mGlu ones are involved in the slow neural response, regulation of neurotransmission, and monitoring of neuronal behaviors (5-8). 

The mGlu receptors are subdivided into three categories based on their structural similarity of amino acids and pharmacological and transduction properties, including mGluRI, II, and III (5-8). These three subgroups of mGluR, act differently based on their G protein properties and their mediated intracellular signaling. The group I receptors are predominantly localized post-synaptically and facilitate excitatory signaling. In contrast, both group II and III mGlu receptors are expressed pre-synaptically and post-synaptically and often dampen post-synaptic excitation either by inhibition of neurotransmitter release or by negatively coupling to adenylyl cyclase activity (8, 9). 

When the release of glutamate exceeded the normal limit due to over-driving of the auditory system, the “excitotoxicity” will take place (10-12). In which, the glutamate changes to be a neurotoxin and disrupts the pre- and post-synaptic elements, which leads to permanent synaptic disorder. As this pathology did not affect auditory thresholds at all, it was not considered important in many clinical settings. However, as this disorder affects negatively many aspects of auditory function, any attempt at finding an appropriate intervention for prevention or remediation of this disease is of value.

In line with providing efficient treatment for such a disease, various methods of modulating glutamatergic signaling pathways have been proposed. The majority of these studies incorporated antagonists of some glutamate receptors or caused a reduction in glutamate production (13-16). The disadvantage of these methods was that they may break up many necessary excitatory synapses and destroy the neural communication, so they cannot be applied in a long period of treatment when a high risk of excitotoxicity is possible. To overcome these problems, we turned to a new intervention based on mGlu receptor activity.

Considering the functionality of mGlu receptors, they have exhibited promising therapeutic effects on a variety of brain disorders such as schizophrenia, epilepsy, stroke, and other disorders in which the etiology of the disease was glutamate overproduction (15, 17-19). This, along with abounding of experimental data linking the mGlu receptor stimulation to down-regulation of excitatory synapses, led to the formation of our hypothesis that in cochlear synaptopathy with the same disease mechanism, the stimulation of mGlu receptors might have therapeutic potential. 

To the best of our knowledge, there has been no published study intervening at the mGlu receptor level in the auditory system. So, in the present study, we stimulated mGluRII via a highly selective agonist, LY354740, injected into the guinea pig cochlea, and then protection from noise-induced cochlear synaptopathy was examined through some electrophysiological and histological approaches.

## Materials and Methods

***Animals***

According to previous similar articles, a total of 12 male albino guinea pigs (250–350 g) were participated in this study (3, 4, 15, 19). All of the animals had a positive Preyer reflex and normal ABR thresholds at 4, 6, 8, 12, and 16 kHz (Table 1). They were randomly divided into two equal groups of sham and experimental ones (n=6, each). The experimental group received LY354740 through direct injection into the cochlea. Instead, normal saline was injected into the sham group. Note that all surgical procedures were the same between the two groups. During the study, the animals were housed under controlled temperature (22±2 °C) and lightning (a half-day lightness/ a half-day darkness cycle) with access to a sufficient supply of food and water. 

All procedures were directed in compliance with the animal care guidelines of the Ministry of Health and Medical Education (20), with an accepted ethical code of IR.IUMS.REC.1398.429.

***Drug solution preparation***

Among many agonists of mGluRII, LY354740 was the most selective and potent one which is effective at low concentration and has no impact, neither excitatory nor inhibitory, on other receptors (21). 

Its characters were compared with previously used drugs such as aminoglycoside and dexamethasone to ensure permeability into the RW membrane (22, 23). Since molecular weight and mass of LY354740 were lower compared with these drugs, its penetrance to the RW membrane was considered definite. 

Concerning EC50 of LY354740 and the optimum concentration reported in its brochure and previous studies about local application to other biological systems, we concluded to take a concentration of 200 nm serially diluted in normal saline (8, 9, 21, 23). The volume of one λ of the solution was drawn into a microsyringe and injected into the desired spot. 

***Drug delivery method***

**Figure 1:**
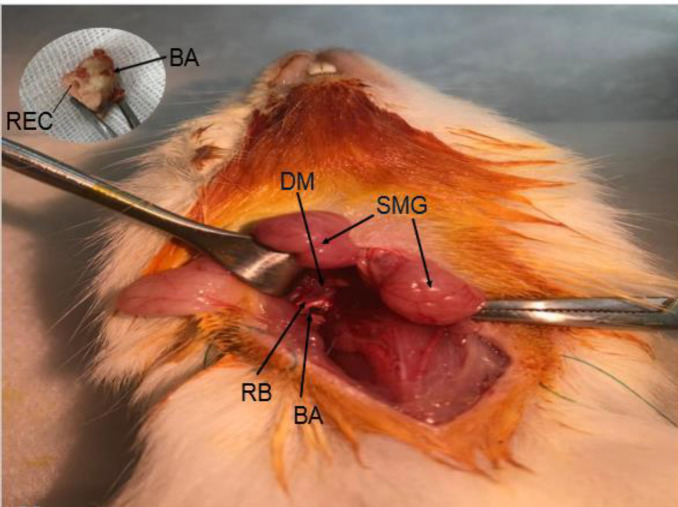
Close view of the surgical area in bullaostomy

A ventral approach was employed to access the bulla of the animals. The animals were located on a sterile surface following deep anesthesia by using ketamine and xylazine (120 and 10 mg/kg, respectively, Alfasan, The Netherlands) in a fixed supine position. The ventral surface of the neck was shaved and the skin cleaned with an antiseptic solution, and subcutaneous injection of epinephrine and xylazine was applied. To get access to the RW of the cochlea, a bullaostomy surgery was performed. Under magnification with a surgical microscope, a 2 cm incision was made longitudinally from mandible to clavicle. The submandibular glands were separated and retracted laterally. Afterward, the digastric muscle with vertical orientation was visible. Following its cutting, the inferior-medial aspect of the tympanic bulla appeared. Figure 1 depicts a close view of a surgical place. A small opening (2 mm) was made using a 27 G needle on the lateral side of the styloid process. The prepared drug solution was injected slowly and directed to the RW niche. The surgical hole was sealed using a small cut of the animal digastric muscle tissue together with a small piece of gel foam. The submandibular glands were returned to their initial position, and the skin was closed with silk surgical sutures.

Ultimately, the animals were kept in a fixed position for about 20 min to ensure the diffusion process and uniformity of drug flow in all parts of the cochlear duct. 

***Noise exposure***

**Figure 2:**
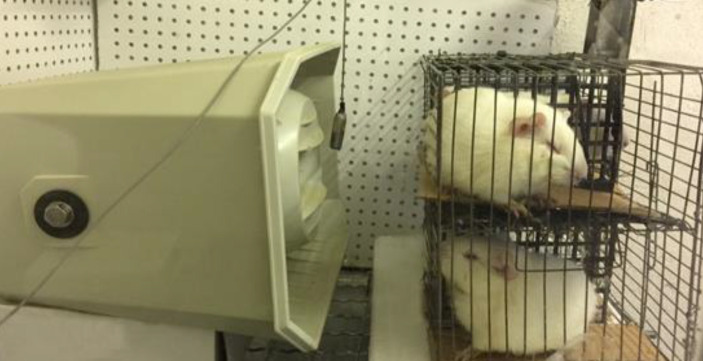
The set-up for presentation of synaptopathic noise to guinea pigs

The animals were exposed to a 2-h noise presentation (104 dB SPL, octave band centered with 4 kHz) generated within a single-walled, sound-deadened chamber. The reason for choosing an octave band noise of 4 kHz was that, considering the physiology of cochlea, the synaptopathic noise will result in a threshold shift mostly in half an octave or an octave above the offending frequency. As our electrophysiological device could assess up to 16 kHz, so the synaptopathic effect of 4 kHz noise could be fully monitored. Additionally, in histological evaluation, the part of the basilar membrane that belonged to the synaptopathic region was completely accessible. The chamber was designed in 60 × 80 × 100 cm of glass and galvanized iron. The noise was built up with a noise generator (Benaphone Electronic Company, Iran) and its level was calibrated with SLM (B & K, model 2243, Denmark). The noise emanated from one speaker equipped with a power amplifier. The speaker was placed in the middle of the roof of the acoustic chamber in order to provide an identical sound pressure level in all parts of the chamber. The distance of the speakers from the ears was 10 cm. During the noise exposure, animals were alert and kept in a wire-mesh cage. Only one animal was placed in a cage to prevent colliding of animals and disruption of intended intensity to reach the animal’s ear (Figure 2).

***Auditory brainstem response measurement***

Animals were anesthetized with an intraperitoneal injection of ketamine 10% (40 mg/kg) and xylazine 2% (4 mg/ kg). Body temperature was maintained at 37.0 ± 0.5 °C using a heating pad. For ABR recording, we incorporated the Bio-logic device (GN Otometrics, Denmark). The electrode array was arranged in a single-channel form with three-needle electrodes placed subcutaneously on the vertex (non-inverted), below the right pinna (inverted), and the contralateral pinna (ground). Interelectrode impedance was kept below 5 kΩ. The stimuli were tone bursts of 4, 6, 8, 12, and 16 kHz presented at a repetition rate of 11.1/sec and with alternating polarity. Responses to 1024 stimuli were preamplified and filtered between 100-3000 Hz. The analysis time of the screen was 10 ms.

The intensity was decreased from 90 dB SPL to the lowest level at which the ABR wave-III, the most stable and repeatable wave in a guinea pig, could be tracked and it was considered as an ABR threshold. 

The peak-to-trough amplitude of wave-I was determined and the amplitude growth curve was obtained by changing the stimulus level from 90 to 40 dB SPL in 10 dB steps. 

For recording masked ABR, tone bursts of 4, 6, 8, 12, and 16 kHz at a constant level of 80 dB SL (re: threshold of that frequency) were presented simultaneously with a broad spectrum noise extended from 1 to 16 kHz. Masking noise was swept from 0 to 80 dB SL (re: threshold of that frequency). The level of noise was initially set at 0 dB SL and it was considered as the no-noise condition then it was increased in 10 dB steps until ABR was just masked and no more response could be recorded. At each noise level, the wave-III latency was analyzed and the amount of its shift compared with the no-noise condition was calculated. 

***Time protocol of the study***

All of the intended tests were repeated in prearranged time points. First, they were done on pre-exposure to noise in order to delineate the baseline values. Then on one day post-exposure (1DPE) for determining the effect of temporary threshold shift (TTS), and after recovering the TTS on one-week post-exposure (1WPE), for confirming the effect of synaptopathy on the tests’ results and one-month post-exposure (1MPE) to approve the permanency of synaptopathy.

***Histological examination***

At the end of the study, at the 1MPE time point, the animals were decapitated under deep anesthesia using a combination of xylazine and ketamine. Temporal bones of the right side were removed, and the bony shell of the cochlea was dissected out. Then, the round and oval windows were exposed, and the perilymphatic spaces were filled with a 2.5% glutaraldehyde and paraformaldehyde as a primary fixation and kept for 48 hr. For elimination of the fixators, the tissue was bathed 2-3 times with phosphate-buffered saline (PBS). Then, 1% osmium tetroxide was used as a secondary fixation for 1.5 hr. The tissue was dehydrated in acetone (50%, 70%, 90%, and 100%) and infiltrated with resin, and finally embedded in pure resin (Epon 812, TAAB, UK). 

The basilar membrane belonging to the 4-8 kHz region was sectioned for TEM (at around 1.5 turns of the cochlea or 10.08-12.65 mm from the apex, based upon the frequency-distance mapping of a guinea pig) (22, 24). Cochlear tissue was sectioned parallel to the modiolar axis. First, a semi-thin (500 nm) section was prepared and contaminated with toluidine blue to make the targeted synaptic part detectable during light microscopy (40–100x). The part of a specimen that belongs to the contact of IHC to auditory nerve terminals was considered as a synaptic zone and used for further consecutive dissections. Second, thin (50 nm) serial sections were performed for electron microscopy. Thin sections were transferred to the 200-mesh uncoated grids and stained with uranyl acetate and lead citrate before imaging by TEM (LEO 906; Zeiss).

***Statistical analysis***

SPSS software, version 16 (Chicago, IL, USA) was incorporated for statistical calculation. Firstly, the normal distribution of the data was analyzed by calculating the standardized skewness and kurtosis index, in the Shapiro-Wilk test. The statistics varied from −3 to 3 with a *P*-value of more than 0.05, suggesting that data distribution was normal. To compare values between pre- and post-exposure time points within each group and also for comparison between groups, ANOVA with repeated measures test was used. In all instances, the significance level was defined as *P*-value≤0.05. 

## Results

***Noise exposure***

**Figure 3:**
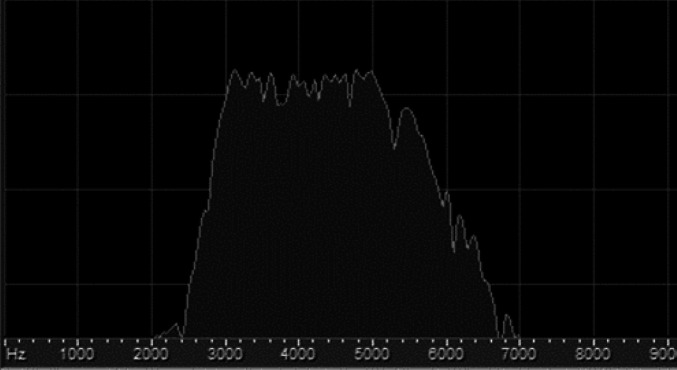
Spectrum of the synaptopathic noise, an octave band noise centered at 4 kHz with dispersion of energy in 3-6 kHz

According to the bulk of experimental works on noise-induced synaptopathy in rodents, it was demonstrated that TTS of approximately 40 dB is most probably comorbid with a definite amount of synaptopathy (11, 25-27). So, in the pilot stage, we used a titration of intensity to reach our expected TTS (40 dB). Finally, by exposure to an octave band noise of 4 kHz, for 2 hr at 104 dB SPL, the expected result was resulted, which was fully recovered in one week follow up. Consequently, the aforementioned noise was presented to the sham and experimental groups to constitute synaptopathy. The spectrum of the noise is shown in Figure 3. 

***ABR thresholds***

Table 1 demonstrates ABR thresholds of 4, 6, 8, 12, and 16 kHz in our sham and experimental groups. The thresholds (mean ± SD) of baseline (pre-exposure), 1DPE, 1WPE, and 1MPE were assessed and the comparison of within groups and between groups was done. 

The ABR thresholds of 4, 6, and 8 kHz were increased in 1DPE with the greatest amount of threshold shift (about 40 dB) at 6 kHz. And threshold shifts were recovered in 1WPE and 1MPE in the sham group. This trend was the same in the experimental group, too. Since the thresholds at 12 and 16 kHz showed no apparent shift in post-noise assessments in either group, we excluded those two frequencies from our further analysis.

There were no significant differences between sham and experimental groups in either frequency, considering the trend of auditory threshold shift (*P*≥0.05).

***ABR wave-I amplitude growth function***

**Figure 4:**
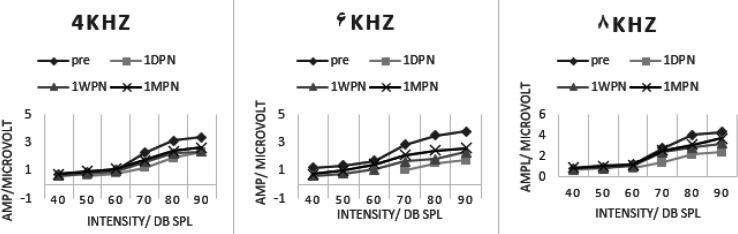
ABR wave-I amplitude growth cure in 4-8 kHz tone bursts in the sham group

**Figure 5:**
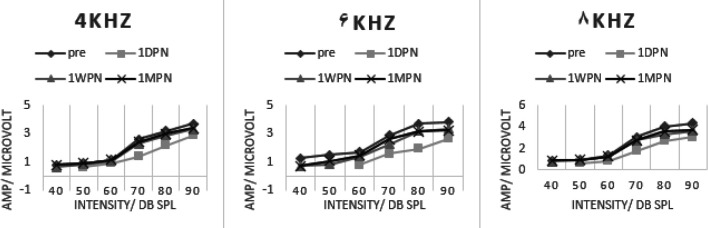
ABR wave-I amplitude growth curve in 4-8 kHz tone bursts in the experimental group

Figures 4 and 5 present the amplitude growth curve of ABR wave-I in the sham and experimental groups. The largest amount of amplitude reduction due to noise exposure was noted in 1DPE, in the sham group. It was followed by a partial recovery in 1WPE assessment; however, there was almost no more significant recovery in 1MPE. The biggest amount of amplitude reduction was observed at 6 kHz, the frequency with the most recorded amount of TTS. The comparison of amplitude at 90 dB SPL in pre-exposure (3.81±0.34 µV) with 1DPE (1.77±0.30 µV) revealed an amplitude reduction to 53.54% that recovered to 61% in 1WPE (2.32±0.54 µV) and showed a little more recovery in 1MPE (2.61±0.24 µV) to 69%. The process of amplitude growth was different in the experimental group. The slope of the growth curve in post-exposure time points was more than that of the sham group. For example, the amplitude at the highest intensity (90 dB SLP) at 6 kHz revealed a reduction to 69.30 % in 1DPE. It recovered to 87.93 % and 85.04% in 1WPE and 1MPE, respectively. 

A repeated-measure ANOVA was performed against pre- and all three post-noise exposure assessments in each group and also between groups. In the sham group, there was a statistically significant difference between pre-exposure and 1DPE amplitude at all intensities in 4, 6, and 8 kHz (*P*≤0.05). However, in the 1WPE assessment, the amplitude increased due to partial recovery, and there were no statistically different values at low intensities. But the differences persisted and weren’t recovered at 70 dB SPL and above even at 1MPE, suggesting the permanency of this abnormal finding. 

In the experimental group, the comparison of the amplitudes between pre- and post-times revealed significant results in 1DPE that resolved mostly in 1WPE and 1MPE. Only at 6 and 8 kHz and at the highest intensities (90 dB SPL) the significant differences persisted in post-noise exposure time points. The between-groups comparison yielded significant differences at 4, 6, and 8 kHz in 70–90 dB SPL. 

***ABR wave-III latency shift in noise***

Masked ABR was recorded for all tone bursts and response detectability was tracked in the presence of different levels of background noise. At the noise level below 30 dB SL, there was no apparent effect of masking noise on the latency. However, the latency began to increase above 30 dB SL. As at 80 dB SL noise level (signal to noise (S/N) ratio of 0), the response was not detected anymore; therefore, the noise at 70 dB SL, set the highest level of our applied masker. 

**Figure 6:**
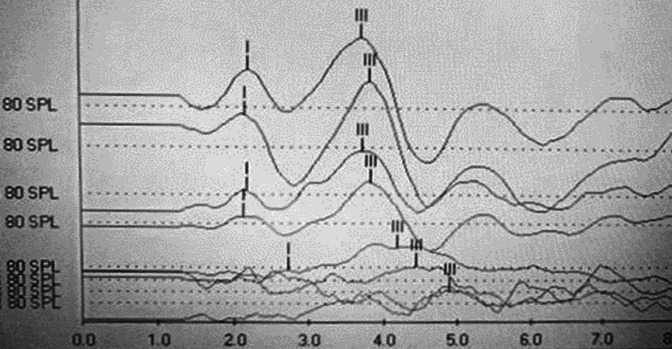
ABR in noise of 6 kHz tone burst at 80 dBSPL and different levels of background noise

Figure 6 depicts ABR in the noise of one case in the pre-exposure assessment. 

The response was detected even at the S/N ratio of 10 in the pre-exposure assessment, meaning that the background noise level till 70 dB SL was tolerable for ABR recording. The presence of tone and noise at the desired SL was not possible at 1DPE due to the severity of hearing loss. So, the test was done just in 1WPE and 1MPE in the sham group. At both of these post-exposure assessments, the response tracked down to 50 dB SL noise level only. The amount of wave-III latency shift in the presence of noise was less than the pre-exposure time point. There were statistical differences between pre- and all the two post-noise exposure assessments of the amount of latency shift of wave-III (*P*≤0.05), with the most significant difference seen at higher noise intensities. *Post hoc* analysis revealed no differences between 1WPN and 1MPN, meaning the permanency of the offending factor during a period of follow-up. 

In the experimental group, the noise tolerance for ABR recording in post-noise exposure times was improved in comparison with the sham group; the masked response could be recorded till the masker of 60 dB SL. Also, the amount of wave-III latency shift in the presence of noise was more than in the sham group. The comparison of within-group values yielded no significant statistic in most instances. There were just significant differences between pre- and post-noise exposure assessments at the highest noise level at each tone burst (60 dB SL). However, between-group comparison revealed significant differences at 4, 6, and 8 kHz at most noise intensities. The related results are depicted in Table 2. 

***Histological findings***

**Figure 7:**
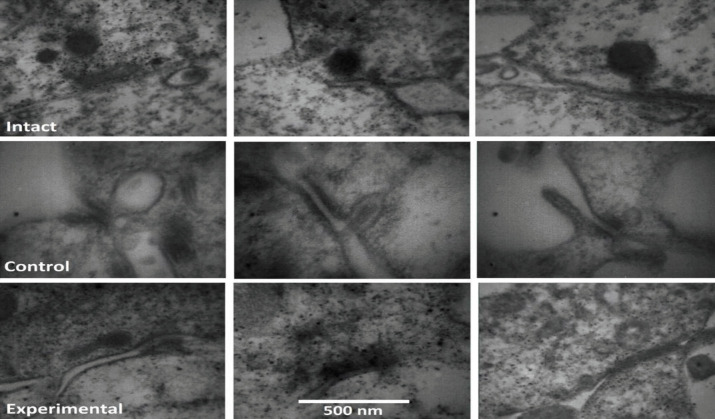
Representative transmission electron micrographs from intact, control, and experimental groups. The electron micrographs showed size, shape of the synaptic ribbon, and postsynaptic density (PSD). In control group compare to intact one, the size of ribbons was larger and hollow and the PSD was thicker and deviated. the results of experimental group was something between control and intact groups

For the final confirmation of synaptopathy, the cochlear tissue of our samples was histologically examined by TEM. The ultra-structural features of synaptic elements of the sham and experimental groups were traced and compared with the intact ones. As shown in Figure 7, the size of the synaptic ribbon in the sham group was found larger than the intact counterpart. This increment of ribbon size was documented by a larger cross-area of ribbon’s existing sections. These swollen ribbons were hollow with less mass density inside, meaning that they lost their intracellular components. It was evident by the brightness of the image on the inner part of the ribbons. The other difference compared with the intact samples was that the ribbons lost their round shape and became spherical with a little irregularity in shape. Additionally, some diversity was also noted in the postsynaptic part. The postsynaptic density (PSD) was thicker in the sham group and missed its normal flat orientation and became deviated.

In the experimental group, the abnormal findings in the histological examination were milder than in the sham group. The ribbon size was something between the sham and the intact groups. The density of the ribbon was more than that of the sham but less than those of the intact ones. Also, the thickness and the deviation of PSD were less than those of the sham group.

**Table1:** Mean ± SD of ABR thresholds in pre-exposure and 3 post noise exposure assessments in sham and experimental groups and their *P*-values

	Pre-Exp Mean ± SD (db)	1DPE Mean ± SD (db)	1WPE Mean ± SD (db)	1MPE Mean ± SD (db)	*p*-Value
Frequency (Hz)	Sham/ Experimental	Sham/ Experimental	Sham/ Experimental	Sham/ Experimental	
**4000**	22.50±2.73 21.66±2.58	45.83±3.76 41.66±2.58	35.83±5.84 30.00±8.36	24.16±2.04 21.66±2.58	0.065
**6000**	22.50±2.73 20.83±2.04	63.66±4.08 60.00±3.16	35.83±12.00 24.16±3.76	24.16±2.04 21.66±2.58	0.086
**8000**	21.66±2.58 20.83±2.04	58.33±6.05 56.66±2.58	32.50±10.08 24.16±7.35	21.83±2.04 22.16±2.04	0.290
**12000**	17.50±2.73 16.66±2.58	24.16±3.76 23.33±4.08	22.50±4.18 23.33±4.08	19.66±3.76 16.66±2.58	0.087
16000	16.66±2.58 17.50±2.73	**20.83±2.04 ** **20.00±5.47**	**19.16±2.04 19.16±3.76**	**16.66±2.58 17.50±2.73**	**0.850**

SMG: Sub mandibular glands; DM: Digastric muscle; RB: Right bulla; BA: Bulla osteomy; REC: Right ear canal

**Table 2:** P-values resulting from comparison of within-group and between groups of ABR wave-III latency shift in noise in different group of animals

		WithinGroup Comparison		BetweenGroup Comparison
		1WPE	1MPE	
Frequency (Hz)	Noise level (dB SL)	*P*-value/ Sham experimental	*P*-value/ Sham Experimental	*P*-value
**4000**	** 30**	0.091 0.271	0.088 0.365	0.175
	** 40**	0.007 0.246	0.007 0.412	0.003
	** 50**	0.000 0.007	0.000 0.009	0.021
**6000**	** 30**	0.040 0.112	0.040 0.276	0.008
	** 40**	0.000 0.112	0.000 0.276	0.000
	** 50**	0.000 0.325	0.000 0.176	0.000
	** 60**	NR 0.005	NR 0.006	
**8000**	** 30**	0.118 0.452	0.122 0.667	0.372
	** 40**	0.000 0.627	0.002 0.691	0.000
50	** 0.000** ** 0.529**	** 0.000** ** 0.618**	** 0.000**

## Discussion

In the current study, the protective effect of a highly selective mGluRII agonist (LY354740) against noise-induced synaptopathy was approved. First, a model of noise-induced synaptopathy was established. It was evaluated by ABR indexes such as growth of wave-I amplitude, latency shift of wave-III, and detectability of response in the presence of noise before and after the intervention. Ultimately, the results were confirmed histologically.

In the present study, the synaptopathy model was designed using a TTS-inducing noise and affirmed to be permanent because it persisted up to one-month follow-up. There was no difference between sham and experimental groups for the threshold of ABR. Since the intervention incorporated in the experimental group had a potential function at the synaptic level and had no protection over hair cells against the exposed noise, that finding was justifiable. Cochlear synaptopathy was initially reported by Kujawa and Liberman in 2009 (28). The deafferentation of synaptic contacts is a hidden pathology that involves the inner ear in presbycusis, ototoxicity, aging, and tinnitus (11). Despite the full recovery of auditory thresholds, the synaptic injury following TTS was permanent in all animal species. (27, 28- 30). So, planning for further understanding of synaptopathy and developing some approach for prevention or treatment is self-evident. 

We observed a reduction in ABR wave-I amplitude following noise-induced synaptopathy in the sham group. The reduction of ABR wave-I amplitude verified synaptopathy in some studies, and oftentimes it didn’t return to its baseline value in spite of threshold recovery (26, 31-34). The underlying neurophysiological basis is the reduction of the amplitude of ABR at high-intensity stimuli due to the injury of low SR neural fibers which are responsible for listening to suprathreshold levels. Actually, in cochlear synaptopathy, the comorbidity of neurotransmitter release was damaged and it prevented the synchronicity of nerve fibers’ response and their temporal summation. So, the growth of amplitude, especially at high intensity was reduced. As the slope of the amplitude growth curve of wave-I in pre-exposure assessment was about 0.56 at 6 kHz which was higher than the values at 1DPE, 1WPE, and 1MPE (0.34, 0.39, and 0.41, respectively); it means that the pre-noise exposure assessment revealed a steeper wave-I growth curve, and the growth of amplitude at a high stimulus level is reduced after noise exposure.

Interestingly, this pattern of amplitude growth function persisted even after accounting for TTS, suggesting that although the partial restoration of malfunctioned synapses is enough for recovery of auditory thresholds, these survived synapses are not adequately proper for higher auditory function in which more precious morphology of synapses is necessarily needed. The reduction of ABR wave-I amplitude was also confirmed in humans, like military veterans, with a history of lifetime noise exposure despite normal auditory thresholds (33, 35).

In the experimental group, the slope of the amplitude growth curve in post-noise exposure was higher than in the sham group. At 6 kHz, the slope of 0.57, 0.60, and 0.57 was recorded for 1DPE, 1WPE, and 1MPE, respectively. These results indicated that LY354740 held a capacity to protect against a synaptopathic noise and prevent amplitude reduction, especially at high stimulus intensity. So, the severity of synaptopathy was less than that of the sham group.

The shift of wave-III latency in masked ABR was reduced in the sham group. Also, the detectability of the response was reduced and it can be recorded just at low levels of noise. It means that high SR fibers were intact after the synaptopathy process act. Simply as they have a restricted dynamic range and saturate at a very low level of background noise, they were not suitable for hearing in noise function. Mehraei *et al*. (2016) found a significant relationship between the amount of latency shift and perceptual temporal processing in humans and reported a smaller slope of wave IV latency in mice with noise-induced synaptopathy (34). According to the findings of the previous studies and that of the present one, the application of masked ABR as a useful metric in synaptopathy diagnoses is defensible. Additionally, in the present study, the detectability of ABR in presence of noise was evaluated for the first time. 

In the experimental group, the response detectability in the presence of noise was improved to 60 dB SL, and the amount of latency shift during masked ABR was more than in the sham group. The differences between our two study groups warranted the regulatory role of LY354740 at the synaptic level that could relatively prevent synaptic loss. It can be concluded that the agonist of mGluRII, LY354740, has the potential to partially protect from noise-induced synaptopathy. The possible mechanism is that along with stimulation of mGluRII in the cochlea, a series of the process that alleviates synaptic injuries through reduction of glutamate release from pre-synaptic elements would be formed. Moreover, it possesses some post-synaptic effects like inhibition of those intra-cellular signalings which were formed due to the accumulation of extra calcium in cells as a result of overdriven glutamate receptors.

Finally, the ultra-structural properties of synapses were further observed. The ribbons were distinguished as a regular, round, and dense structure in the control group with no noise exposure or intervention. However, in the sham group, the ribbons appeared hypertrophic and spherical resembling an empty view. Since the process of glutamate synthesis is a calcium-dependent phenomenon, in over-stimulation of a system for providing added glutamate, the amount of intracellular calcium should be increased. The accumulation of calcium led to volume increment of the ribbon. Additionally, the aggregation of excessive calcium in the cell initiated a deleterious calcium-dependent signaling pathway with harmful byproducts that led to the death of intracellular elements and made the ribbon seem hollow inside. Another study by Song *et al*. (2016) also reported a larger ribbon size at the modiolar side of the cochlea which belongs to low SR fibers, low-density space inside the ribbon, swollen post-synaptic terminals, increase of synaptic vesicles, and deviation of PSD angle (36). We also noted some structural changes in post-synaptic parts, including the thickening of PSD and deviation of its angle. In the experimental group, the abnormal findings in the histological examination were less than in the sham group. These findings suggest that concomitant to our electrophysiological results, the histological findings revealed fewer synaptic injuries in the experimental group. This finding supported the protective role of LY354740 against noise-induced synaptopathy.

To the best of our knowledge, there has been no published study about mGluR activation in auditory disorders. However, some studies have investigated the role of this receptor in the prevention and treatment of other neurological diseases. Chaki (2010) reported alleviation of schizophrenic symptoms following the application of mGluRII agonist. They related this therapeutic effect to the reduction of glutamate release from the prefrontal cortex (17). Yao *et al*. (2015) cited the inhibitory effect of mGluRII activation on excessive cell proliferation in the dentate gyrus of a rat model of epileptic seizures through some intracellular signaling mediated by the mGluRII’s G protein (15). Aligned with these studies, in another study done in 2017, Chandrasekaran and colleagues reported the reduction of the symptom of peripheral neuropathy in a rat model of diabetes following the application of mGluRII agonist. They attributed this neuroprotective property of the receptor agonist to stimulation of the associated chain of SIRT1 and maintenance of mitochondrial OXOHOS function (18). In another recent study in 2018, the effect of mGluRII activation on the protection of oxidative stress in rat brains of hypoxia-ischemia model was evaluated. They noted that soon after stimulation of this receptor, the neuroprotective mechanism formed that reduced the oxidative stress and ROS (19). Another study showed alleviation of symptoms in cases of anxiety and drug withdrawal by application of mGluRII (37, 38). The common property of all the mentioned studies was that the protective role of mGluRII on these diseases was along with prevention of excessive glutamate release or inhibition of glutamate-dependent negative intracellular events. Similarly, in overstimulation of the auditory system, the same destructive process due to extra glutamate production will occur.

## Conclusion

The higher slope of the ABR-wave I amplitude, the more tolerance of noise in masked ABR, concomitant with the histological finding that revealed less synaptic damage, confirmed the therapeutic effect of LY354740 in cochlear synaptopathy. In line with studies about mGluRII activation for prevention or treatment of excitotoxicity, in the present study for the first time, we provided evidence that agonists of this receptor have the potential to protect from cochlear synaptopathy. 

## Authors’ Contributions

MP, AP, VPM and MRA Designed and performed the experiments. MP, AP and MK Collected the data. MP, AP and AKY Discussed the results and strategy. MP Directed and managed the study. Final version to be published was approved by all.

## Conflicts of Interest

The authors declare that no conflict of interest exists.
